# Implementation Challenges of Community Directed Treatment with Ivermectin Program for Control of Onchocerciasis in Ulanga, Tanzania

**DOI:** 10.24248/eahrj.v5i2.661

**Published:** 2021-11-15

**Authors:** Vivian Mushi

**Affiliations:** a Department of Parasitology and Medical Entomology, School of Public Health and Social Sciences, Muhimbili University of Health and Allied Sciences, Dar es Salaam, Tanzania

## Abstract

**Background:**

Community drug distributors (CDDs) have a crucial role in distributing ivermectin for onchocerciasis control and prevention. Their roles, experiences and challenges faced in the implementation of community-directed treatment with ivermectin (CDTI) programme could potentially affect coverage, consequently leading to persistent transmission. Therefore, this study aimed to explore the experience and the roles which CDDs plays in implementation of community directed treatment with ivermectin program for onchocerciasis control in Ulanga, Tanzania.

**Methods:**

A cross-sectional study design was used to collect qualitative data in 2018 in Ulanga district, Tanzania. Five community drug distributors were purposively selected for in-depth interviews. Thematic framework approach for qualitative data analysis was used to generate codes, categories and themes.

**Results:**

Out of the five community drug distributors interviewed, two had experience of 15 to 20 years on implementation of the community directed treatment with ivermectin programme while the remaining community drug distributors had experience of less than 10 years. The main challenges faced by CDDs in the implementation of the CDTI programme include; the geographical location of the hamlets (hard to reach hamlets), long distances between houses, low compliance of community members to medication due to fear of side effects experienced before and mistrust of methods of dose calculation, short time of drug distribution and absence of people from their households as the exercise was conducted when community members were involved in agricultural activities.

**Conclusions:**

The challenges faced in the implementation of the CDTI programme could negatively affect the distribution and coverage of ivermectin treatment in the Ulanga district. It's now an opportune time to address the challenges that CDDs are facing in the implementation of the CDTI programme to ensure effective control of onchocerciasis in the district.

## BACKGROUND

Onchocerciasis is commonly called River blindness after its geographic locus and most visible symptom. Overall, it causes blindness, disfigurement, and unbearable itching in victims, while rendering large tracts of farmland uninhabitable. This chronic parasitic disease affects skin and eyes. The causative agent for onchocerciasis is a parasitic filarial worm *Onchocerca volvulus*, of the family *filaridae*. Onchocerciasis is transmitted by the bite of infected black flies^1^ of the genus *Simulium*. Black flies breed in fast flowing streams and rivers because of the demand for highly oxygenated water during the maturation of the larvae. Females require a blood meal for ovulation, and they transmit infective-stage (3^rd^ stage) larvae as well as ingest microfilariae during the blood meal. The black fly tends to stay within 2 km of its breeding site. The adult worms pair and mate in the human host, and unlike most nematodes that produce eggs, the female *Onchocerca* gives birth daily to thousands of microscopic larvae known as microfilariae. Those microfilariae migrate to tissues and induce inflammatory reaction when they die, and also, the parasite causes visual impairment and irreversible blindness.^2^

The recent World Health Organization (WHO) data reported that 198 million residents from 31 countries in Sub-Saharan Africa and five countries in the Americas and Arabian Peninsula are at risk of acquiring onchocerciasis. In 2017, 20.9 million people were infected with onchocerciasis, and among the infected people, 14.6 million had skin diseases, and 1.15 million had vision loss.^3,4^ In Tanzania, 6 million people are at risk of infection in seven foci, namely; Kilosa, Mahenge, Morogoro, Ruvuma, Tanga, Tukuyu, and Tunduru.^5^ The history shows that Mahenge's focus where Ulanga District is located carries the highest burden of onchocerciasis since the 1990s, with the microfilariae prevalence of around 60% to 87% and nodule prevalence of 95%.^6^

The burden of onchocerciasis in Tanzania led to the introduction of CDTI in 1997 by the African Programme for Onchocerciasis Control.^5,6^

The CDTI is the main intervention for onchocerciasis control using community members known as CDDs for ivermectin delivery in the communities.^7^ The CDTI programme has proven to be successful in reducing onchocerciasis transmission and morbidity when applied annually at coverage of at least 80% within 12 to 15 years.^8,9^ However, the persistent transmission of onchocerciasis has been reported in some areas after 17 to 20 years of annual CDTI.^10,11^ Community drug distributors play a crucial role in the implementation of the CDTI programme, they consist of volunteers selected by community members to distribute ivermectin.^12^ Community drug distributors are trained and re-trained every 1 to 2 years to deliver drugs in the community and educate community members on health issues.^13^

In Ulanga district where this study was conducted in Tanzania, a persistent transmission of 2.9% was observed after two decades of annual CDTI.^14^ This was supported by the presence of 0.57% infected black fly vectors from the same area indicating that the two decades of annual CDTI have not interrupted transmission.^15^ Community drug distributors plays significant roles in ensuring the success of the CDTI programme. However, challenges faced by CDDs in implementing the CDTI programme have not been fully explored and mitigated. The challenges could be a hindrance to effective implementation of the programme hence elimination of the disease. Therefore, this study aimed to explore the implementation challenges encountered by CDDs and assess how these challenges impacted program outcomes in Ulanga, Tanzania.

## METHODS

### Study Design and Setting

A community-based cross-sectional study involving qualitative methods of data collection was carried out in Ulanga district between June and July 2018. This study was a part of the large study that was conducted in Ulanga district in June and July 2018. Ulanga was selected because of persistent transmission of onchocerciasis despite more than two decades of CDTI programme implementation.

Ulanga district is among the six districts of the Morogoro region located at latitude of −8°59’19.90” S and longitude of 36°36’47.92” E. The district has an area of 24,460 km^2^ with an approximate population of 169,853 whereby females are 85,098 and males are 84,755.^16^ The main economic activities in Ulanga are subsistence farming, fishing, and mining.

The district has one hospital, two health facilities, and 21 dispensaries. In health services, the following are diseases present; onchocerciasis with nodding syndrome, epilepsy, malaria, worms (soil-transmitted helminths), anemia, diarrhea, pneumonia, protein-calorie malnutrition, tuberculosis, acute Respiratory Infection, diabetes, HIV/AIDS, hypertension, and asthma.^17^

The district experiences tropical climatic conditions characterised by annual rainfall approximately between 800mm and 1600mm every year. Also, the district is characterised by perennial rivers that support the survival and breeding of *Simulium damnosum* s.I.^11^

### Study Population

Study population included all 5 key informants (CDDs) from two villages (3 from Uponera village and 2 from Isongo village). Community drug distributors consist of volunteers, both women, and men selected by community members, for distributing ivermectin without payment. Therefore, they have their main jobs for earning income to support their lives.

### Sampling

The study used a multi-stage sampling technique to obtain the representative villages. The first stage involved a random selection of the two representative wards from Ulanga district whereby Uponera and Isongo were selected. The second stage involved a simple random selection of one representative village in each of the selected wards whereby Uponera and Isongo were selected. In each of the selected villages, the total number of CDDs was obtained. Uponera village had three CDDs, and Isongo village had two CDDs hence, all of them were invited to participate in this study.

### Data Collection

An interview guide was prepared by the researcher and had five sections. The first section collected information on the socio-demographic characteristics of respondents while the second section was composed of questions on experience, selection and training of CDDs. The third section was composed of questions on participation, distribution of drugs and coverage of the CDTI programme. The fourth section was composed of questions on challenges they were facing during the implementation of the CDTI programme and the last section had questions on improvement of the CDTI programme. The interviews were conducted in Kiswahili language and were audio taped. Short notes were also taken to ensure that all the questions and responses were properly recorded.

### Data Analysis

The collected data from in-depth interviews were analysed qualitatively using a thematic framework approach. The collected audio data were transcribed verbatim in Kiswahili to obtain the textual format then translated to the English and thereafter back into Kiswahili. The data were coded manually and then organized into categories. Finally, analysis and interpretations were done by clustering similar and related topics together to form major themes as presented into results section.

### Ethical Consideration

Ethical clearance was sought from the Muhimbili University of Health and Allied Sciences Ethical Review Board (Ref. No. DA. 287/298/01. A/). Permission to conduct the study in the Ulanga district was requested from the Regional Administrative Secretary of Morogoro, then-District Administrative Secretary, and District Medical officer of Ulanga district after submitting introduction letter attached with ethical clearance from MUHAS. Community drug distributors were informed about the study, given informed consent forms, and requested to sign if they were willing to participate in the study. Community drug distributors were assured that no one will be harmed in the participation of the study and a high level of confidentiality was maintained in the study whereby numbers were used instead of names of participants. All audio recorded data were transferred into the personal computer of the researcher and locked with a password so that nobody else could access the data.

## RESULTS

### Socio-demographic characteristics of the study respondents

A total of 5 CDDs were interviewed, their ages ranged from 25 years to 70 years. All CDDs interviewed were self-employed and residents of the Ulanga district as shown in Table 1.

**TABLE 1:** Socio-Demographic Characteristics of Study Respondents (n=5)

Characteristics	n (%)
**Gender**	
Male	2(40)
Female	3(60)
**Age group**	
25–35	1(20)
36–45	-
46–55	3(60)
>56	1(20)
**Level of Education**	
Primary school education	4(80)
Secondary education	-
College/University education	1(20)
**Occupation**	
Employed	-
Self-employed (Peasants)	5(100)
**Duration of Residence**	
25–35	1(20)
36–45	-
46–55	3(60)
> 56	1(20)

### Theme one: Experience, selection and training of CDDs

Out of the 5 CDDs interviewed, four of them were appointed by the village executive officer and members of the village committee because of their experience in community services and hard work except for only one who volunteered. This shows that community members in Ulanga did not have a chance to select their CDDs as recommended by the WHO regulations.

The results also showed that only two CDDs had worked in their position for 15 to 20 years and the remaining ones for less than 10 years, as revealed in the following statements;

*“………I have been working as a volunteer CDD for 15 years; I volunteered to work in this position because at that time no one wanted to work in this position”* (Female respondent, Uponera, 55 years).

*“……..I was a nurse assistant at the time CDTI programme started in 1997 people used to come and take ivermectin at the district hospital, so when the distribution of drug started directly in the community I was appointed by village executive officer to help my community because of my experience”* (Female respondent, Isongo, 49 years).

When the CDDs were asked and probed on type of the training that was given for their position, all of them stated that they were trained every year before the distribution of the ivermectin. One of the CDDs, for example had this to say:

*“…….The training is given once every year to remind each other on how to distribute drugs, the measurement to be taken to know the exact dose taken by a person, and how to manage side effects as a result of treatment”* (Male respondent, Uponera, 70 years).

### Theme two: Community participation, distribution and coverage on CDTI programme

Community drug distributors were asked to state how they distributed ivermectin and how do they ensured that community participated in CDTI programme. The results showed that *“house to house distribution*” was the main approach that was employed to distribute ivermectin and when it comes to participation of the community in the programme the CDDs said they had the following roles;

“…….*After taking the medication from the district hospital, I must announce to community members, emphasize them to take medication and then I distribute the drugs* from *one house to another in the entire hamlets*” (Female respondent, Uponera, 55 years)

*“…….In the past community members used to collect medication at my house, but now I must pass house to house so as to ensure people take medication and if people are not there I must come back or leave the message for them to come to collect the medication”*(Male respondent, Uponera, 70 years).

The key informants indicated that women's uptake of ivermectin was higher than men's as confirmed by one of the CDDs;

“…….*Women highly participate in the control programme compared to men except those who are sick, pregnant or have delivered within five days at the time of drug distribution”* (Male respondent, Isongo, 51).

### Theme three: Challenges faced by CDDs on implementation of CDTI programme and recommendation for improvement

Community drug distributors were asked to state the challenges they were facing during ivermectin distribution because these challenges were thought to affect the use of ivermectin in the community and hindered effective success of CDTI programme to control the onchocerciasis disease. The mentioned challenges were mainly geographical related to the location of hamlets. Some of the hamlets were hard to reach (located in a remote and not easily accessible), and this led to the failure of CDDs to cover all houses, long distances between houses made the CDDs walking extra miles and spent more days distributing the ivermectin. Another challenge was low compliance of community members to medication due to fear of side effects experienced before and mistrust of methods of dose calculation. Absence of people from their houses was another challenge, as in Ulanga many community members were migratory farmers. The distribution of the drug for control of onchocerciasis was often conducted during farming season when many community members were involved in agricultural activities. Another challenge was duration of drug distribution, the time allocated for drug distribution was short and CDDs were required to return the ivermectin to the health centers after a month. The following are some of the responses as revealed by two CDDs:

*“…… There are several challenges that I face as a CDD. Some community members are refusing to take medication because of side effects so its wastage of time going to a certain house and talking to them and at the end they are refusing to take medication. Also, the geographic location of our village is the problem because houses are far from each other so it's difficult to reach every house”* (Female respondent, Isongo, 49 years).

Another CDD mentioned transport to be a critical problem;

“……*Transport is the problem in our village which makes the distribution to be difficult hence some of the houses are left unattended. The number of days for distribution of medicines are few that we are supposed to return the remaining medicines to district hospital after a month”* (Male respondent, Isongo, 51 years)*.*

On recommendations for improving the CDTI programme, the CDDs gave the following recommendations as a strategy to mitigate the challenges faced in the implementation of the CDTI programme. The distribution of drugs should be done after the farming season when people are at home and are free to participate. This will improve the participation of the migratory farmers in the CDTI programme. Transport fees should be provided to assist the CDDs to reach all villages at times of ivermectin distribution. Instead of a one-day training (current practice), key informants recommend three-day training to enable them comprehend the concepts. Health education about onchocerciasis should be given to community members at least once a year to avoid misconceptions about the effects of the treatment. Finally, all CDDs recommended allowance to be given to them to increase their morale for work as they were not benefiting anything from being CDDs and the work was very difficult.

## DISCUSSION

Community drug distributors have a significant role to ensure the community participates in CDTI programme. It is important for community to be careful when selecting CDDs because they can influence, motivate and educate the community to participate in CDTI programme.^11,18^

However, in Ulanga community members were not involved in the selection of CDDs, the majority of them were appointed by village executive officer with exception of one CDD who volunteered, the practice which is inconsistent with WHO regulations.^8^ It was observed that some of the appointed CDDs were highly experienced, this is because they had been working in this position for a long time since the beginning of the CDTI programme hence have undergone several short trainings. Community drug distributors in Ulanga were trained every year on how to distribute drugs properly in the community so that could educate community members on the importance of ivermectin treatment. Clearly, these findings are similar to the findings of the study conducted in North-Western Ethiopia on knowledge, attitudes and practices of CDDs about onchocerciasis and CDTI.^13^

Distribution of ivermectin in Ulanga was through house to house, the practice which is in accordance with WHO guidelines.^8^ Community drug distributors in Ulanga have a role to educate, motivate and ensure that the community members participate in the control of onchocerciasis which was done through households visits before ivermectin distribution to sensitize the community. Despite the efforts of the CDDs, the coverage of CDTI in Ulanga is fluctuating, ranging from less than 65% in 1997 to 2002, and more than 65% (mean 76%) for the years 2003 to 2017, being below the optimal coverage of 80%. ^5^ Some of the barriers to sufficient coverage as mentioned by CDDs included; poor timing of drug distribution as most of the time distribution is done during farming seasons where people are away from their houses, insufficient time allocated for drug distribution as CDDs are required to return the ivermectin to the health centers after a month, hard to reach hamlets due to their geographic location and hence CDDs fail to cover all houses, doubt on the method of dose calculation and fear of side effects occurring as a result of taking ivermectin.

Of the mentioned challenges by CDDs, hard to reach hamlets, doubt on the method of dose calculation, and fear of side effects were also revealed by another study done in Tanzania.^19^ Side effects of ivermectin were among the major barriers of community participation in the CDTI program in Ulanga because when a community member experiences side effects or sees their fellows experience side effects may negatively affect uptake in the coming year due to worries, consequently causing fluctuations in coverage. Though the CDDs in Ulanga are trained, they lack the confidence to handle side effects because the community does not trust them, as previously documented in Morogoro, Tanzania.^19^ Hence, the recommendation of the provision of health education to the community is crucial to resolve the ongoing misconception on ivermectin treatment.

With regard to gender; women were highly participating in ivermectin control programme compared to men especially when they saw their fellow women as CDDs. This is in contrast with a study conducted in the previous year's which showed men as the head of the households were highly participated in the CDTI programme compared to women, and most of the CDDs were males.^20^ Recently, in Tanzania, female CDDs have shown to be more tolerant and patient than men.^21^ Similarly, in Uganda, for example, about 70% of the community members believed that women were more persuasive, committed, and patient compared to men when it came to ivermectin distribution activities hence, improving the compliance of community members towards treatment.^22^

### Study limitations

The study had the following limitations; the CDDs were required to remember the previous year's information on their selection, training, and coverage of the ivermectin distribution hence, the high possibility of recall bias. Also, the study relied on the CDDs reported experiences and challenges rather than direct observation from the study area.

## CONCLUSIONS

Community drug distributors have a critical role in ensuring the success of the CDTI programme for onchocerciasis control in Ulanga. The low experience of some CDDs coupled with the challenges they are facing in the implementation of the CDTI has the potential to affect total uptake and coverage of the CDTI programme. Therefore, as we are aiming to eliminate onchocerciasis by 2030, now more than ever it's the opportune time to address the ongoing challenges so as to improve and sustain coverage of the CDTI programme in Ulanga district. Based on the CDDs' insights, there is a need for a neglected tropical disease control programme (NTDCP) to revise the existing guidelines for CDDs. The NTDCP should set the qualification and criteria of the selection of the CDDs, revising the training programme to add to the health education component, and mitigating the challenges faced by the CDDs. Also, the NTDCP should consider the provision of incentives to boost the working morale of the CDDs.
